# Comparison of whole genome sequences from human and non-human *Escherichia coli* O26 strains

**DOI:** 10.3389/fcimb.2015.00021

**Published:** 2015-03-11

**Authors:** Keri N. Norman, Michael L. Clawson, Nancy A. Strockbine, Robert E. Mandrell, Roger Johnson, Kim Ziebell, Shaohua Zhao, Pina M. Fratamico, Robert Stones, Marc W. Allard, James L. Bono

**Affiliations:** ^1^U.S. Meat Animal Research Center, United States Department of Agriculture, Agricultural Research ServiceClay Center, NE, USA; ^2^Division of Foodborne, Waterborne and Environmental Diseases, National Center for Emerging and Zoonotic Infectious Diseases, Centers for Disease Control and PreventionAtlanta, GA, USA; ^3^Western Regional Research Center, United States Department of Agriculture, Agricultural Research ServiceAlbany, CA, USA; ^4^Laboratory for Foodborne Zoonoses, Public Health Agency of CanadaGuelph, ON, Canada; ^5^Division of Animal and Food Microbiology, Center for Veterinary Medicine, Food and Drug AdministrationLaurel, MD, USA; ^6^Eastern Regional Research Center, United States Department of Agriculture, Agricultural Research ServiceWyndmoor, PA, USA; ^7^Food and Environment Research AgencySand Hutton, York, UK; ^8^Division of Microbiology, Center for Food Safety and Applied Nutrition, Office of Regulatory Science, Food and Drug AdministrationCollege Park, MD, USA

**Keywords:** *Escherichia coli*, O26, Shiga toxins, polymorphisms, phylogenetic

## Abstract

Shiga toxin-producing *Escherichia coli* (STEC) O26 is the second leading *E. coli* serogroup responsible for human illness outbreaks behind *E. coli* O157:H7. Recent outbreaks have been linked to emerging pathogenic O26:H11 strains harboring *stx*_2_ only. Cattle have been recognized as an important reservoir of O26 strains harboring *stx*_1_; however the reservoir of these emerging *stx*_2_ strains is unknown. The objective of this study was to identify nucleotide polymorphisms in human and cattle-derived strains in order to compare differences in polymorphism derived genotypes and virulence gene profiles between the two host species. Whole genome sequencing was performed on 182 epidemiologically unrelated O26 strains, including 109 human-derived strains and 73 non-human-derived strains. A panel of 289 O26 strains (241 STEC and 48 non-STEC) was subsequently genotyped using a set of 283 polymorphisms identified by whole genome sequencing, resulting in 64 unique genotypes. Phylogenetic analyses identified seven clusters within the O26 strains. The seven clusters did not distinguish between isolates originating from humans or cattle; however, clusters did correspond with particular virulence gene profiles. Human and non-human-derived strains harboring *stx*_1_ clustered separately from strains harboring *stx*_2_, strains harboring *eae*, and non-STEC strains. Strains harboring *stx*_2_ were more closely related to non-STEC strains and strains harboring *eae* than to strains harboring *stx*_1_. The finding of human and cattle-derived strains with the same polymorphism derived genotypes and similar virulence gene profiles, provides evidence that similar strains are found in cattle and humans and transmission between the two species may occur.

## Introduction

Shiga toxin-producing *Escherichia coli* (STEC) O26:H11/NM has emerged as an important human pathogen capable of causing severe cases of diarrhea and hemolytic uremic syndrome (HUS) (Misselwitz et al., 2003). Also, enteropathogenic *E. coli* (EPEC) O26 strains lacking Shiga toxins (*stx*) have been heavily implicated in cases of infantile gastroenteritis (Zimmerhackl et al., 2010). The true incidence of STEC O26 infections is largely underrepresented because most laboratories lack standardized methods for detecting non-O157 STEC strains. Current research has largely focused on developing reliable methods for detecting non-O157 strains in clinical samples, food animals, and animal products (Verstraete et al., 2012; Wang et al., 2012; Kalchayanand et al., 2013; Windham et al., 2013; Yonekita et al., 2013). Current data indicates that STEC O26:H11/NM are the second most common STEC serotypes responsible for infections following O157:H7 (Gould et al., 2013).

Outbreaks of STEC infection have been traced to the O26 serogroup in the United States (Brown et al., 2012), Europe (Werber et al., 2002; Ethelberg et al., 2009; Buvens et al., 2011), and Japan (Misselwitz et al., 2003; Sonoda et al., 2008; Tomita, 2008). The most common serotype implicated in outbreaks is O26:H11 (Kaspar et al., 2010). Several of the more recent O26:H11 outbreaks include one in a Colorado childcare center in 2011 (Brown et al., 2012), a 2007 ice cream implicated outbreak in Belgium (De Schrijver et al., 2008), and a 2007 outbreak in Denmark traced to beef sausage (Ethelberg et al., 2007). The 2011 outbreak in the childcare center in Colorado was the largest reported O26 outbreak in the United States with 18 confirmed cases and an additional 27 suspected cases (Brown et al., 2012). More recently in 2012, 29 individuals from 11 states were infected with an O26 STEC strain traced back to clover sprouts (Centers for Disease Control and Prevention, 2012).

*E. coli* O26 strains may contain a variety of virulence factors including Shiga toxin genes (*stx*_1_, *stx*_2_, or both *stx*_1_ and *stx*_2_), the enterohemolysin gene *ehxA*, and/or the *eae* gene that encodes for intimin involved in attachment. Human pathogenic O26 strains containing *stx*_2_ initially emerged in Germany in the 1990s (Zhang et al., 2000), but have become increasingly reported in the United States (Misselwitz et al., 2003; Brooks et al., 2005) and Europe (Allerberger et al., 2003; Käppeli et al., 2011). It has been hypothesized that O26 strains carrying *stx*_2_ have increased virulence and are associated with an increased risk of HUS, in comparison to strains that are predominant in cattle and only contain *stx*_1_ (Bielaszewska et al., 2013). The reservoirs for these emerging STEC O26:H11 *stx*_2_ strains are still largely unknown; however strains have been isolated from healthy domestic ruminants in Switzerland (Zweifel et al., 2013).

STEC O26 has been isolated from the feces of cattle, sheep, goats, and pigs and also has been detected in animal-derived meats (Hussein and Sakuma, 2005; Dambrosio et al., 2007) and dairy products (Steele et al., 1997). In addition to non-pathogenic *E. coli* O26 strains, cattle have been shown to harbor STEC and EPEC O26 strains that may cause serious disease in humans (Jeon et al., 2006; Sasaki et al., 2012; Paddock et al., 2013). In fact, STEC O26:H11 was among the top 10 most common STEC serotypes isolated from cattle and beef products in Canada (Johnson et al., 1996). The finding of STEC *E. coli* O26 in the feces of food animals and in animal products is a concern regarding potential foodborne transmission of this pathogen. STEC, EPEC, and enterohemorrhagic *E. coli* (EHEC) O26 strains from humans and food animals have been previously compared using pulsed-field gel electrophoresis (PFGE), multi locus sequence typing (MLST) and whole genome sequencing. Leomil et al. (2005) found that human and cattle strains were closely related and shared similar genotypic characteristics. Ju et al. (2012) showed that the genomes of all O26:H11 strains sequenced were similar and at least one human isolate clustered with several cattle and one swine strain using whole genome sequencing and MLST.

The objectives of this study were to identify unique nucleotide polymorphisms in a panel of human and non-human derived O26 *E. coli* strains in order to compare differences in polymorphism derived genotypes and virulence gene profiles between the two host species. The majority of the non-human strains were of bovine origin. The DNA from the human and non-human strains were pooled separately and sequenced for single nucleotide polymorphism (SNP) discovery. A set of SNPs was selected for MALDI-TOF genotyping and resulting polymorphism-derived genotypes were used to construct phylogenetic trees. Genotypes were compared to evaluate relationships between serotypes, virulence genes, and host both within and between phylogenetic clusters.

## Materials and methods

### Strains

A total of 291 *E. coli* O26 strains were included in this study for sequencing, genotyping, or both; 182 were classified as human strains and 109 as non-human. Non-human strains included 84 bovine, seven strains of unknown origin, six avian, three fly, three water, two goat, two sheep, one antelope, and one feral hog. Serotypes represented among the strains included 259 O26:H11, 20 O26 strains with unknown H types, 7 O26:NM, 3 O26:H32, 1 O26:H36, and 1 O26:H46. The *stx* gene profiles consisted of 210 *stx*_1_, 15 *stx*_2_, 18 both *stx*_1_ and *stx*_2_, and 48 non-STEC. Other virulence genes included 233 that carried both *ehxA* and *eae*, 40 *eae* only, 1 *ehxA* only, and 17 neither *ehxA* nor *eae* (Supplemental Table 1). The strains came from various geographic locations worldwide, including 223 from the United States, 40 from Canada and one each from Mexico, Australia, France, and Switzerland. Strains were characterized by PCR for Shiga toxin (*stx*_1_ and *stx*_2_), intimin (*eae*), hemolysin (*ehxA*), O-antigen (*rfb*_O26_), and flagellar (*fliC*_H11_) genes (Paton and Paton, 1998; Durso et al., 2005).

### DNA extraction, O26 DNA pools, and 454 GS FLX sequencing

Two genomic DNA pools were created for sequencing. One pool contained DNA from 109 human-derived strains and the second pool contained DNA from 73 non-human-derived strains [59 bovine, three fly, three water, two sheep, two unknown origin, one antelope, one avian, one feral swine, and one goat (Supplemental Table 1)]. Among the sequenced strains there were 182 STEC (151 *stx*_1_ only, 15 *stx*_2_ only, and 14 both *stx*_1_ and *stx*_2_) and two non-STEC strains (Supplemental Table 1). Genomic DNA was extracted using the Qiagen Genomic-tip 100G (Qiagen, Valencia, CA) with modifications as previously described (Clawson et al., 2009). The DNA pools were constructed by adding 3 μg of DNA per strain into their respective pools, i.e., human or non-human. Genomic sequencing libraries were created from each pool using the Roche GS FLX Titanium Rapid Library Preparation Kit (Roche Diagnostics, Indianapolis, IN) followed by emulsion PCR according to the manufacturer's protocol and run using a Roche Genome Sequencer GS-FLX+. To prepare reads for mapping, sequencing reads were filtered using the Roche GS FLX Data Processing Software. For nucleotide polymorphism genotyping, Qiagen QIAamp DNA mini extraction kits were used to isolate genomic DNA according to the manufacturer's directions for an additional 109 strains [73 human, 25 cattle, five strains of unknown origin, four chicken, one goat, and one turkey (Supplemental Table 1)]. Among 109 strains that were genotyped but not part of the DNA pools for sequencing, there were 63 STEC (59 *stx*_1_ only and 4 both *stx*_1_ and *stx*_2_) and 46 non-STEC strains (Supplemental Table 1).

### Nucleotide polymorphism discovery and genotyping

Nucleotide polymorphisms were identified by mapping 454 GS FLX sequencing reads from the human and non-human pools onto the chromosome sequence of STEC O26:H11 strain 11368 (Ogura et al., 2009) using Geneious Mapper (Biomatters Ltd., Auckland, New Zealand). After the initial identification, a reduced number of nucleotide polymorphisms was selected from the two pools for subsequent genotyping of individual strains. These polymorphisms were selected based on their minor allele frequencies in the pools and their locations in the reference genome that were likely to be conserved amongst all strains of the O26 serogroup. The identified nucleotide polymorphisms were used by the MassARRAY® assay design software to design matrix-assisted laser desorption ionization-time-of-flight (MALDI-TOF) assays and multiplexing as recommended by the manufacturer (Sequenom, Inc., San Diego, CA). The software designed primers for multiplex PCR primer (Up to 36 polymorphisms into an individual assay) and the ensuing probes for genotyping. The assays were run with iPLEX Gold® chemistry (Sequenom, Inc.) on a MassARRAY® genotyping system per instructions of the manufacturer.

### Identification of tagging nucleotide polymorphisms

For 289 of 291 O26 strains used in this study, the alleles of 283 nucleotide polymorphisms were genotyped and concatenated to produce polymorphism-derived genotypes. A ClustalW alignment of the polymorphism-derived genotypes was produced in MacVector (Version 12.0.6). From that alignment, a minimal set of polymorphism-derived genotypes that contained a single representative of every unique polymorphism-derived genotype was identified in Tree-Puzzle (version 5.2). Putative tagging nucleotide polymorphisms were then identified from the minimal set of polymorphism-derived genotypes using the Tagger option of Haploview (version 4.2) with *r*^2^ = 1, and with the option to capture all pairwise alleles through pairwise tagging. The putative tagging nucleotide polymorphisms were then systematically subtracted; one SNP at a time, from Clustal W alignments, and Tree-puzzle was used to check for redundant sequences as previously described (Bono et al., 2012) to identify a minimal set of tagging polymorphisms.

### Phylogenetic analyses

Neighbor-Joining trees were produced from Clustal W alignments of full-length nucleotide polymorphism-derived genotypes, and those defined by the minimal set of tagging polymorphisms in PHYLIP (version 3.69). The trees were made using an F84 distance model of substitution with a transition/transversion ratio of 2, and were bootstrapped with 1000 pseudo-datasets. The trees were viewed using Treeview (version 1.6.6). Additionally, a Neighbor-joining network was constructed from a Clustal X alignment of a minimal set of tagging polymorphisms using SplitsTree4 (version 4.12.3). Maximum likelihood trees were constructed with the Tamura-Nei model, uniform rates among sites, and with gaps deleted. The search was performed with SPR level 5 and very strong branch swapping settings in MEGA (version 6) (Tamura et al., 2013). Parsimony analysis using TNT (version 1.1) included a new technology search with the sectorial search, ratchet, drift, and tree fusing options all enabled, with the minimal length tree found 10 times (Dereeper et al., 2008, 2010).

### Pulsed-field gel electrophoresis

PFGE was performed on 35 epidemiologically unrelated, human-derived O26 strains that were sequenced and genotyped. Thirty-one of the strains were O26:H11 and the remaining four were O26:NM. The strains had similar virulence gene profiles; 33 *stx*_1_, *eae*, and *ehxA*; 1 *stx*_1_ and *eae*; 1 *stx*_1_, *stx*_2_, *eae*, and *ehxA*. PFGE followed methods previously published for *E. coli* O157:H7 and utilized the restriction enzyme *XbaI* (Ribot et al., 2006; Bono et al., 2012). Data were analyzed using Bionumerics Software (Applied Maths, Austin, TX) and compared to gel images provided by the Centers for Disease Control and Prevention. The strains were categorized by PFGE banding patterns and these categories were compared to the polymorphism-derived genotypes.

## Results

### Sequencing coverage of O26 strains

A total of 1.233 GB of sequence was produced in the study, with 591 MB originating from the pool of 111 human strains, and 642 MB originating from the pool of 71 non-human strains. Given that the size of the STEC O26 strain 11386 chromosome (Ogura et al., 2009) is approximately 5.7 Mb, the mapped depth of coverage per sample for the human pool was 0.9X (103.7X total for the pool) and 1.5X (112.6X total for the pool) for the non-human pool.

### Identification and validation of polymorphisms

Mapping sequencing reads onto STEC O26 strain 11386 chromosome (Ogura et al., 2009), using Geneious mapper, identified a total of 32,710 nucleotide/indel polymorphisms with a minor allele frequency of 0.05 or higher (data not shown). From these, 12,229 polymorphisms mapped to prophages, insertion sequences, transposable elements and other repetitive regions in the STEC O26 chromosome and were removed from consideration for SNP validation. Repetitive regions create problems for SNP validation because it is difficult to determine whether sequences that are virtually indistinguishable from one another result from a true variation at a single site or result from multiple copies of a gene in the genome. Also, plasmids were not used due to lack of conserved sequences between strains. Of the remaining 20,481 polymorphisms that placed within more conserved regions of the STEC O26 chromosome, 8864 were identified in the human pool and 11,617 in the non-human pool.

MALDI-TOF assays were developed for 342 polymorphisms that were chosen for validation according to their minor allele frequency in the DNA sequencing pools and genomic position. MALDI-TOF genotyping of the polymorphisms across 289 O26 strains yielded 29 polymorphisms with heterozygous genotypes or more than 10% no calls in addition to 30 monomorphic polymorphisms that were all removed from further analysis. A total of 283 polymorphisms were validated by MALDI-TOF genotyping of which 226 reside in open reading frames with 134 predicted as non-synonymous or premature stop codon allele variants (Supplemental Table 2). From the 283 polymorphisms, the minor alleles of 17 polymorphisms were observed exclusively in one of the DNA pools at a frequency of 15% or higher with eight in the human pool and seven in the non-human pool. For polymorphisms lower than 15% but no lower than 5%, 24 were in the human pool and 62 in the non-human pool. Additionally, 180 polymorphisms were observed in both pools where 84 had a minor allele frequency greater than 15% and 49 with a minor allele lower than 15%, but not lower than 5%. The remaining 47 polymorphisms had an allele frequency greater than 15% in only one of the pools.

### Identification of polymorphism derived genotypes in *E. coli* O26 strains

**Figure 1:**
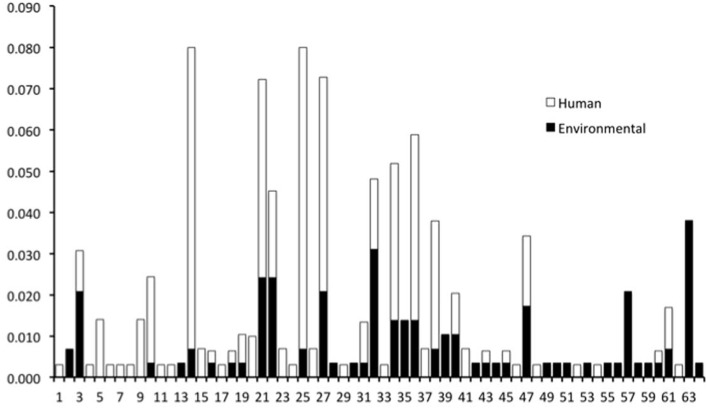
**Frequency of human and non-human-derived strains for the 64 polymorphism-derived genotypes**.

**Figure 2:**
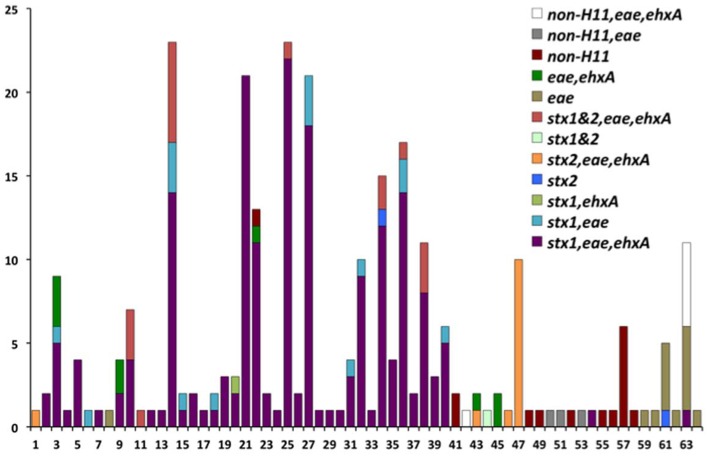
**Virulence gene profiles within the 64 polymorphism-derived genotypes. Each bar or stacked bar represents the number of strains with the virulence gene profile per genotype**.

The genotypes of 289 O26 strains (241 STEC and 48 non-STEC) were concatenated from 283 validated polymorphisms and yielded 64 unique polymorphism-derived genotypes (Supplemental Table 3). The 64 genotypes could be derived from a minimal set of 43 polymorphisms (Supplemental Table 4). From these, 24 genotypes were exclusively found in human-isolated strains, 19 exclusively from the non-human sources, and 21 were found in both human and non-human strains (Figure 1, Supplemental Table 1). Genotypes 14 and 25 had the highest frequency (21 strains) of STEC O26 human strains while genotype 32 had the highest frequency (nine strains) of STEC O26 non-human strains. The 15-*stx*_2_ only strains were placed in six genotypes (1, 34, 43, 46, 47, and 61) and were the exclusive *stx* profile in three of those genotypes (1, 46, and 47) (Supplemental Table 1). Of the strains that carried *stx*_2_ only, 66.7% fell within genotype 47 (Figure 2). The 210-*stx*_1_ only strains were placed in 39 different genotypes with the highest frequency (22 strains) in genotype 25 followed by genotypes 21 and 27 (21 strains each). The 18 strains with both *stx*_1_ and *stx*_2_ strains were placed in eight different genotypes with the highest frequency (six strains) in genotype 14. Genotypes 14 and 25 had the highest frequency (23 strains) of US strains while genotype 21 had the highest frequency (nine strains) of Canadian strains. The 48 non-STEC strains were placed in 24 different genotypes with the highest frequency (10 strains) in genotype 63 followed by genotype 57 (six strains).

### Phylogenetic analysis of O26 polymorphism-derived genotypes

**Figure 3:**
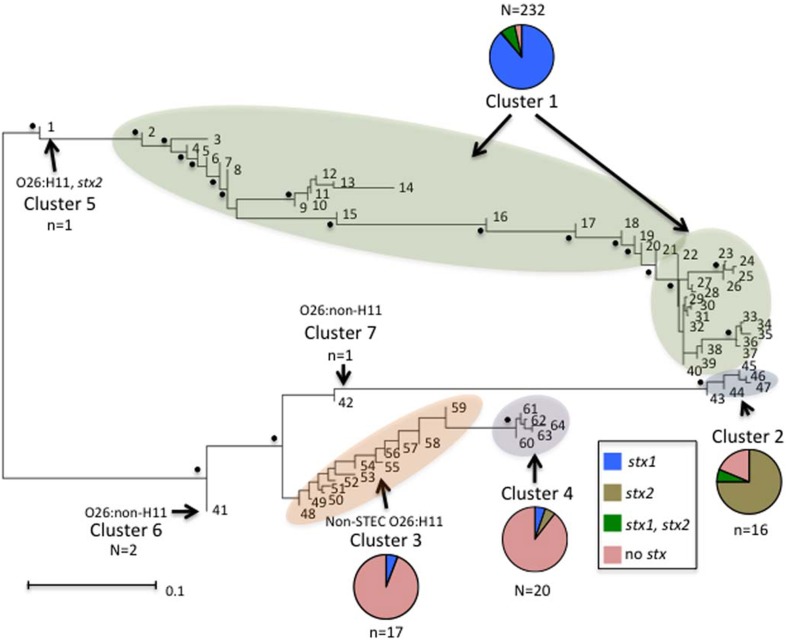
**Neighbor-joining tree of the 64 full-length polymorphism-derived genotypes. Bootstrap values greater than 80 are indicated by a black dot. Outer taxonomic unit numbers correspond to the polymorphism-derived genotypes**. The seven clusters are indicated and clusters containing multiple genotypes are highlighted. The frequencies of the different Shiga toxin profiles from each cluster are represented by pie charts with colors representing the different profiles. The scale bar represents substitutions per site.

**Figure 4:**
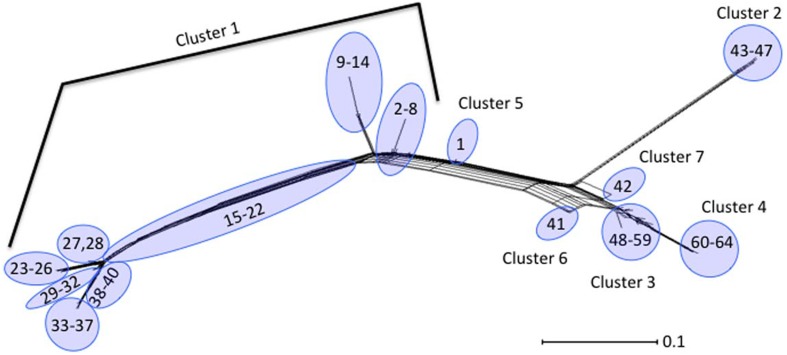
**Neighbor-Joining network of the 64 polymorphism-derived genotypes based on all 283 polymorphism alleles**. Outer taxonomic unit numbers correspond to the polymorphism-derived genotypes.

Neighbor-Joining trees were constructed from the 64 polymorphism-derived genotypes that were produced from (1) all 283-polymorphism alleles, and (2) a minimal set of 43 tagging polymorphisms (Figure 3, Supplemental Figure 1). Of the two trees, the one representing 283 polymorphism alleles contained higher overall bootstrap support and was used for cluster identifications. Seven clusters were defined within the tree by the phylogenetic relatedness of the genotypes, and by the presence or absence of Shiga toxin genes (*stx*_1_ or *stx*_2_), *eae, ehxA*, and/or H11 serotype (Figure 3). The clusters were represented by as few as one genotype (Clusters 5, 6, and 7) and as many as 39 different genotypes (Cluster 1). Five of the seven clusters (1–2, 4–6) were well supported by bootstrap values, which indicated strong support of their placement in the tree. These results were supported by maximum parsimony and maximum likelihood analysis with the exception that Cluster 6 was collapsed into Cluster 3 (data not shown). To examine the support for Clusters 3 and 7 without using bootstrap values, a Neighbor-Joining network of the 64 polymorphism-derived genotypes based on all 283 polymorphism alleles was created (Figure 4). This network differed from the tree in that it allowed for recombination events. All seven clusters were defined within the network, and thus were reproduced between Neighbor-Joining trees and networks.

Distinct genetic determinants defined the polymorphism-derived genotypes within each of the seven clusters. Cluster 1 contained 39 genotypes that represented 232 O26 strains that were predominantly O26:H11 (231 strains), *stx*_1_ (223 strains), *eae* (230 strains), and *ehxA* (214 strains) positive and *stx*_2_ negative (214 strains) (Supplemental Table 1). The five genotypes of Cluster 2 represented 16 O26:H11 strains that were predominantly s*tx*_2_ (13 strains), *eae* (15 strains), and *ehxA* (15 strains) positive and *stx*_1_ negative (15 strains). Cluster 3 contained 12 genotypes and 17 strains that were represented by 13 O26 non-STEC strains that were *eae, ehxA*, and H11 negative (12 had unknown H type and one was H46), three non-STEC strains of unknown H type that were positive for *eae*, and one STEC O26:H11 strain that was positive for *stx*_1_, *eae*, and *ehxA*. Cluster 4 contained five genotypes that were represented by 20 O26 strains, of which 13 were H11 and *eae* positive and five other strains that were H11 negative and *eae* positive. The remaining two strains in Cluster 4 were positive for *ehxA* and *eae*, with one carrying *stx*_1_ and the other *stx*_2_. Cluster 5 was represented by a single O26:H11 strain that was *stx*_2_, *eae*, and *ehxA* positive. Cluster 6 was represented by two O26 strains that had identical polymorphism-derived genotypes, and identical virulence gene profiles (negative for *stx*_1_, *stx*_2_, *eae, ehxA*, and H11). Lastly, Cluster 7 was represented by a single O26 isolate that was *eae* and *ehxA* positive, and negative for H11. As a whole, both human and non-human strains were represented in the clusters and virulence factor frequencies defined many of the clusters. In that regard, of the 17 strains with polymorphism-derived genotypes that placed in Cluster 3, only three were isolated from humans, which is notable as most of the strains in Cluster 3 were negative for many of the major virulence determinants.

### Comparison of polymorphism-derived genotypes and PFGE

**Figure 5:**
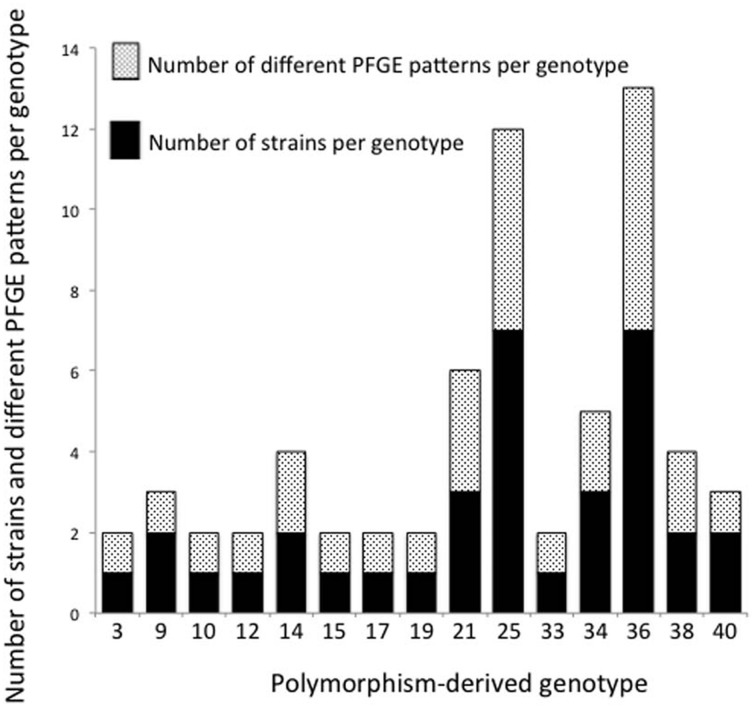
**Number of strains and PFGE patterns in 15 polymorphism-derived genotypes**. Stacked black bars or white speckled bars represent the number of strains and PFGE patterns per genotype, respectively.

Thirty-five epidemiologically unrelated, human STEC O26 strains were compared for genetic diversity based on PFGE and polymorphism-derived genotypes. PFGE profiles were determined using the restriction enzyme *XbaI* and banding patterns assigned using the CDC standard naming protocol. From the 35 strains, there were 26 PFGE patterns and 15 polymorphism-derived genotypes, with multiple PFGE patterns observed within six of the genotypes and seven manifested as singletons (Figure 5). Two genotypes contained the same PFGE profile, while three sets of strains with the same PFGE pattern were classified into different genotypes (data not shown).

## Discussion

We sequenced and analyzed the genomes of 180 STEC and two non-STEC *E. coli* O26 strains from human and non-human sources. We identified 283 nucleotide polymorphisms in conserved regions of the chromosome that were utilized for MALDI-TOF genotyping of 180 sequenced strains and an additional 109 O26 *E. coli* strains. Genotyping data was used to compare polymorphism-derived genotypes and virulence gene profiles among strains from human and other non-human sources, particularly cattle. The concatenated polymorphism alleles identified 64 unique genotypes representing 289 strains that on phylogenetic analysis were grouped into seven clusters. Clusters were not distinguished by host species but did depend strongly on virulence gene profiles.

Similarities between human and cattle strains were found using whole genome sequencing and SNP genotyping. Among the 64 unique SNP genotypes, 21 were found in both human and non-human strains (79% of the non-human strains were of cattle origin) (Figure 1). Previous studies that have used PFGE, MLST, and whole genome sequencing to compare O26 strains from human and food animals have found similar results (Leomil et al., 2005; Ju et al., 2012). Using PFGE and MLST, Leomil et al. (2005) found two genetically distinct groups among 23 *E. coli* O26 strains. One group contained EPEC and EHEC strains from both humans and food animals and the second group contained EPEC strains from both humans and food animals that were phenotypically and genetically distinct from previously characterized O26 strains. Comparing seven O26:H11 strains using whole-genome sequencing, Ju et al. (2012) found that one human strain clustered among four cattle and one swine strain.

We found seven distinct clusters of O26 strains using whole genome sequencing, SNP analysis, and phylogenetics. The clusters were well supported by strong Neighbor-joining bootstrap values and reproducibility between phylogenetic algorithms. A similar whole genome sequencing study and SNP analysis was performed on a set of O26 EHEC strains from Europe (Bielaszewska et al., 2013; Bletz et al., 2013). The SNP genotyping of 120 human pathogenic strains that originated from Germany that harbored *stx*_1_, *stx*_2_ or both *stx*_1_ and *stx*_2_, resulted in 10 unique SNP profiles that phylogenetically grouped into four clonal complexes (clonal complexes shared ≥90% of the SNPs). When comparing our SNPs (Supplemental Table 2) with those of Bletz et al. (2013), we found that only 23 of the 283 SNPs and three of the 43 tagging polymorphisms matched. One reason for this low proportion of matched SNPs may be that O26 strains in Europe and the United States may have undergone phylogeographic stratification. Additionally, strains were isolated from different sources between the two studies; human and non-human STEC and non-STEC strains were sequenced in the current study; whereas, Bletz et al. (2013) focused mainly on virulent human STEC strains. The increased number of clonal complexes in our study may be the result of a larger and more diverse pool of O26 strains.

The phylogenetic clusters with multiple genotypes (1, 2, 3, 4) in our study were represented by both human- and cattle-isolated strains, suggesting that similar strains were found in humans and cattle. Human- and cattle-derived strains were also present in many of the genotypes; notably, 66.7% of our STEC O26 strains carrying only *stx*_2_ were in genotype 47, represented by similar numbers of human and cattle strains. The phylogenetic analysis by Bletz et al. (2013) suggests a source-sink evolutionary model, in that a stable population of O26 strains exists in a niche and only a few strains with evolutionary advantages are transferred to a new niche (Bletz et al., 2013). These new niches may account for the distinct clonal complexes and the emergence of the highly virulent strains of O26 in relevant reservoir and human hosts (Bletz et al., 2013). Our data support the hypothesis that a source population of non-pathogenic O26 strains exists in the environment, and that select strains have established themselves in cattle. These strains may have acquired virulence factors either before or after establishing in cattle as a result of selective pressures. These virulent strains may ultimately be transferred to humans where they are detected through disease.

Human and cattle strains in the current study also had similar virulence gene profiles. Among the 289 O26 strains genotyped, there were *stx*_1_ only, *stx*_2_ only, strains containing both *stx*_1_ and *stx*_2_, and non-STEC strains. There were six genotypes (1, 34, 43, 46, 47, and 61) with *stx*_2_ only strains; three of the genotypes had human and cattle strains (34, 43, and 47), two genotypes contained only human strains (1 and 46), and genotype 61 contained human strains and one strain from an unknown source (Figure 2; Supplemental Table 1). This is interesting because there has been a trend toward *stx*_2_ only O26 pathogenic strains (Zhang et al., 2000). STEC O26 strains from cattle have predominantly carried *stx*_1_ and there have been few reports of the prevalence of *stx*_2_ strains in cattle. Recently, two of 11 STEC O26:H11 strains from healthy cattle in Switzerland contained *stx*_2_ and *eae* (Zweifel et al., 2013). However, further investigation is required to determine if cattle are an important reservoir for the emerging virulent STEC O26 *stx*_2_ strains in humans. The finding of human and cattle strains with the same genotypes, and the similarity of virulence gene profiles between strains in both host species, suggests that transmission of *E. coli* O26 may occur between humans and cattle or both host species are infected by a common source.

Phylogenetic analysis of the genotypes revealed several interesting patterns regarding particular virulence gene profiles. In the neighbor-joining tree in Figure 3, seven clusters of O26 strains on two distinct branches are shown. The majority of O26 strains in Cluster 1 were *stx*_1_ only; however there were also both *stx*_1_ and *stx*_2_, *stx*_2_ only, and non-STEC strains. Cluster 5 was represented by a single O26:H11 *stx*_2_ only strain. Cluster 1 and Cluster 5 were on a distinct branch from Clusters 2, 3, 4, 6, and 7. In Cluster 2, 13 of 15 strains were *stx*_2_ only. All strains but one in Cluster 3 were non-STEC O26 strains. Cluster 4 contained O26 strains harboring *eae* with one *stx*_1_ only and one *stx*_2_ only strains. Cluster 6 contained two non-STEC O26:non-H11 strains, and Cluster 7 contained a single non-STEC O26:non-H11 strain harboring *eae*. This phylogenetic tree of the 64 unique genotypes provides evidence that O26 *stx*_2_ strains evolved separately from O26 *stx*_1_ strains and are more similar to non-STEC O26 strains. Interestingly, Bletz et al. (2013) also found *stx* to be an important virulence factor associated with phylogenetic clustering in O26 strains. Similar to our results, they found that the emerging highly virulent O26 strains harboring *stx*_2_ clustered separately from the other strains and suggested that a source-sink model is responsible for the distinct clonal complexes. However, there were few non-pathogenic strains included in this study so it is difficult to assess the phylogenetic relationship between the *stx*_2_ strains and non-STEC strains in comparison to the phylogenetic relationship between *stx*_2_ strains and *stx*_1_ strains. In regards to the source-sink model, our data would suggest that *stx*_1_ and *stx*_2_ strains have followed distinct evolutionary paths that may have been determined by differing selective pressures or fitness of the strains.

Several strains included in this study were among numerous diarrheagenic *E. coli* (DEC) strains previously genotyped using multilocus enzyme electrophoresis (Whittam et al., 1993). Across all the DEC strains, there were a total of 191 electrophoretic types (ETs) identified; however 15 ETs accounted for 70% of the strains in the study (Whittam et al., 1993). Twenty ETs were found among 93 O26 strains (Whittam et al., 1993). Four DEC9 and five DEC10 strains were included in our study. While most DEC9 strains lack *stx* and have *eae* and the H32 flagellar antigen (Whittam et al., 1993), those in our study were all O26:H11, but *eae* positive and *stx* and *ehxA* negative. On the other hand, the DEC10 strains typically were all O26:H11 and *eae* and *ehxA* positive, and three of the five were *stx*_1_ positive. Our results agreed with this classification scheme. The five DEC10 strains were classified in genotypes in Cluster 1, which were generally O26:H11 strains with *eae* and *stx*_1_ (Supplemental Table 1). The four DEC9 strains on the other hand, were classified in genotypes in Cluster 4, which were generally O26:H11 strains with *eae* but no *stx*. When looking at the 15 most common ETs associated with all the *E. coli* strains, Whittam et al. (1993) found DEC9 and DEC10 strains clustered separately, and although they are more closely related to each other than to other *E. coli* serogroups, they do have distinct evolutionary lineages. We also found that DEC9 and DEC10 strains had distinct evolutionary lineages. DEC10 strains were found in Cluster 1, which were on a separate branch of the phylogenetic tree from DEC9 strains in Cluster 4 (Figure 3).

A comparison of SNP derived genotypes with PFGE data for 35 epidemiologically unrelated, human-derived strains found a higher diversity among PFGE patterns (Figure 5). There were 26 PFGE patterns and 15 genotypes identified among the strains. Within the 15 genotypes, six contained multiple PFGE patterns. It was previously determined that SNP derived genotypes do not surpass the diversity provided by PFGE patterns in a study of 96 epidemiologically unrelated STEC O157 strains from ground beef (Bono et al., 2012). Bono et al. (2012) identified a total of 68 PFGE patterns using restriction enzymes *XbaI* and *BlnI*, and 41 polymorphism-derived genotypes were found using whole-genome sequencing. Within the 41 genotypes, 17 genotypes contained two or more PFGE profiles (Bono et al., 2012). Although these studies of STEC O26 and O157 provide evidence that PFGE provides better discriminatory resolution than polymorphism-derived genotypes for studying genetic diversity, PFGE does not provide data on the evolutionary relationships between strains.

In summary, whole genome sequencing and SNP-based phylogenetic analysis of *E. coli* O26 strains in this study revealed that human and cattle strains did not cluster in separate polymorphism-derived genotypes, and with some exceptions, they had similar virulence gene profiles. These findings suggest that similar strains are circulating between humans and cattle. In regards to virulence gene profiles, we found that strains harboring only *stx*_1_ clustered separately from strains harboring only *stx*_2_, strains harboring *eae*, and non-STEC strains. This would suggest that the emerging strains harboring only *stx*_2_ may have evolved separately from *stx*_1_ strains and are more closely related to non-STEC strains. The full extent of animal and non-human reservoirs for O26 with *stx*_2_ is unknown, however our findings suggest that cattle may be a reservoir for STEC O26 carrying *stx*_2_ that are emerging in humans. Further investigation of a larger collection of human and non-human STEC O26 strains with only *stx*_2_ beyond the 15 strains in this study, combined with other STEC O26 and *stx* variants is required to better identify their reservoirs and define their evolution.

### Conflict of interest statement

The authors declare that the research was conducted in the absence of any commercial or financial relationships that could be construed as a potential conflict of interest.

## Supplementary material

Supplemental Figure 1 **Neighbor-joining tree of polymorphism-derived genotypes tagged with a minimal set of 43 polymorphisms**. Bootstrap values greater than 80 are indicated by a black dot. The scale bar represents substitutions per site.
