# Supplementary figures and images for: Comparison of whole genome sequences from human and non-human Escherichia coli O26 strains
Source: Front Cell Infect Microbiol. 2015 Mar 11;5:21. doi: 10.3389/fcimb.2015.00021 (PMC4356229; doi:10.3389/fcimb.2015.00021)

Supplemental Figure 1

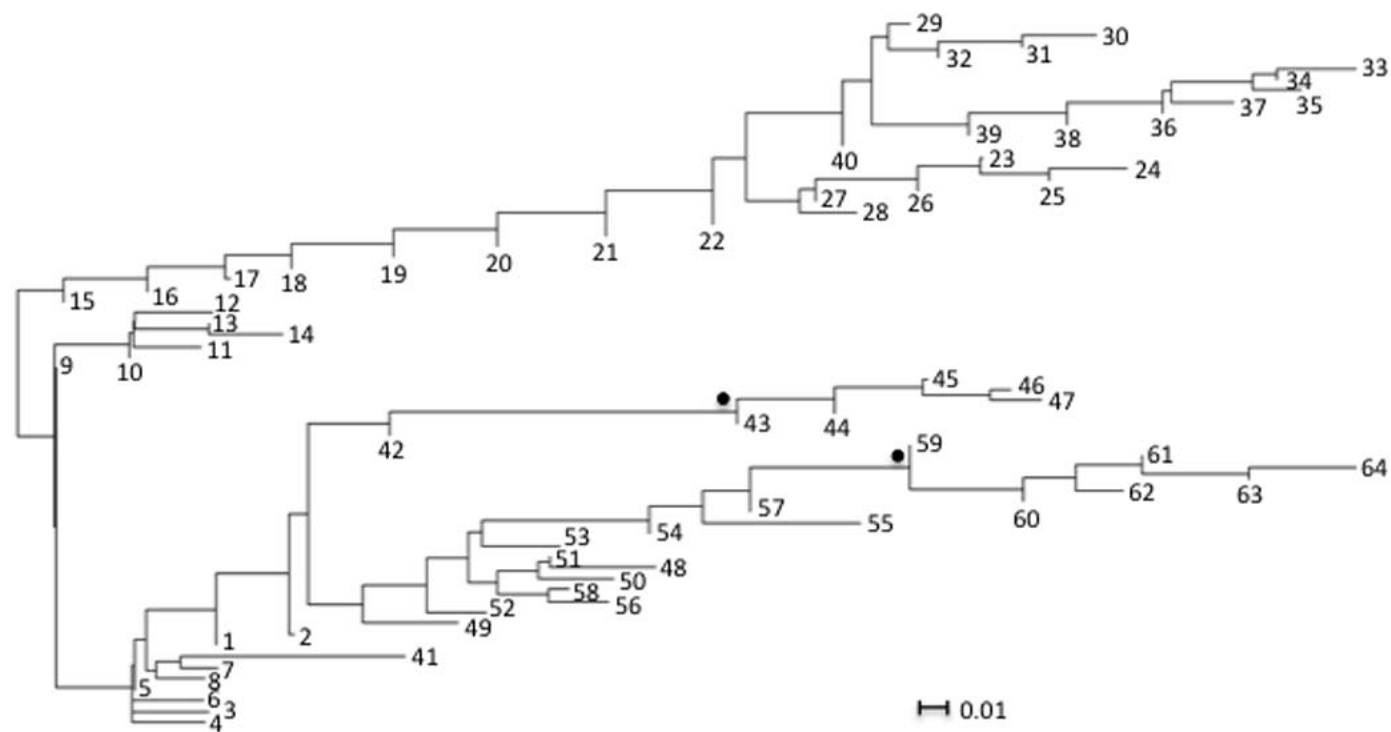

Supplement: Supplemental Figure 1 — Neighbor-joining tree of polymorphism-derived genotypes tagged with a minimal set of 43 polymorphisms. Bootstrap values greater than 80 are indicated by a black dot. The scale bar represents substitutions per site. [file Image1.PDF]
